# Acknowledgement to Reviewers 2025

**DOI:** 10.3389/ijph.2026.1609823

**Published:** 2026-04-27

**Authors:** IJPH Editorial Office

**Affiliations:** Swiss School of Public Health (SSPH+), Zürich, Switzerland

The Editors of the *International Journal of Public Health* would like to thank all Reviewers in 2025 for the time they have spent and the valuable contributions they have made to ensure the scientific quality of the journal.

Charles Abongomera, Switzerland

Philip Abughul, Switzerland

Laura Débora Acosta, Argentina

Bosede Odunola Adejugbe, United States

Shrief Afifi, Egypt

Priya Agarwal-Harding, United States

Claudia Agnoli, Italy

Yogesh Ahire, India

Usoro Akpan, United Kingdom

Fatme Al Anouti, United Arab Emirates

Abdallah Al-Ani, United States

Bea Albermann, Switzerland

Yusuf Alhaji Surakat, Czechia

Karim A. Mohamed Al-Jashamy, Malaysia

Josue Almansa, Netherlands

Lubna A. Alnasser, Saudi Arabia

Mohammed Alsabri, United States

Zeynep Meva Altaş, Türkiye

Flavia Alves, Brazil

Donato Angelino, Italy

Iluta Arbidane, Latvia

Stefania Arena, Italy

Tetty Rina Aritonang, Indonesia

Uğur Ata, Türkiye

Goncalo Augusto, Portugal

Adriana Smaranda Baban, Romania

Maria Bacikova-Sleskova, Slovakia

Elena Baixauli, Spain

Karthik Balajee, India

Damiano Baldassarre, Italy

Hailey Banack, Canada

Massimiliano Barattucci, Italy

Cassandra Barbey, France

Tess Bardy, Switzerland

Georg F. Bauer, Switzerland

Esra Eren Bayindir, Türkiye

Martina Behanova, Austria

Dritan Bejko, Luxembourg

Ivanka Bekavac Vlatkovic, Croatia

Lisa Berg, Sweden

Luan Vinicius Bernardelli, Brazil

Marlene Joannie Bewa, United States

Saurav Bhattacharya, United States

Yogesh Bhosale, India

Lukas Blinka, Czechia

Giovanna Boccuzzo, Italy

Stefan Boes, Switzerland

Gary Bond, United States

Alberto Borraccino, Italy

Aylene Bousquat, Brazil

Dara Bracken-Clarke, United States

Francesca Bracone, Italy

Beatrice Braut, Italy

Elizabeth Breeze, United Kingdom

Julia Browne, United States

Jose A. Calvache, Spain

Antonio Augusto Carioca, Brazil

Flávia Emília Cavalcante Valença, Italy

Marina Cavalieri, Italy

Shobana Chandrasekar, India

Lena Charlette, India

Rachel P. Chase, United States

Ming-Shu Chen, Taiwan

José Chen-Xu, Colombia

Paolo Chiodini, Italy

Patricia Chocano-Bedoya, Switzerland

Yun-Hee Choi, Canada

Marco Matteo Ciccone, Italy

Tiago Correia, Portugal

Jean Tenena Coulibaly, Côte d'Ivoire

Zsombor Csata, Romania

Stéphane Cullati, Switzerland

Adam Czabański, Poland

Andrea Dalecká, Czechia

Elizabel De Souza Ramalho Viana, Brazil

Jean-Marie Degryse, Belgium

Tafadzwa Dhokotera, Switzerland

William D'Hoore, Belgium

Kaisheng Di, China

Augusto Filippo Di Castelnuovo, Italy

Rasit Dinc, United States

Pasquale Dolce, Italy

Jose L. Domingo, Spain

Keren Dopelt, Israel

Małgorzata Drywień, Poland

Daniel Du Plooy, Germany

Tatiana Dubayová, Slovakia

Mari Dumbaugh, United States

Mehmet Nuri Duran, Türkiye

Tamer Edirne, Türkiye

Marloes Eeftens, Switzerland

Hande Erman, Türkiye

Clémence Esse, Côte d'Ivoire

Monica Ewomazino Akokuwebe, South Africa

Maria Luisa Farnese, Italy

Michael Feldhaus, Germany

Chuanteng Feng, China

Alessandro Feraldi, Italy

Romulo Fernandes, Brazil

Daniela Filakovska Bobakova, Slovakia

Maddalena Fiordelli, Switzerland

Alice Fognani, Italy

Mario Fordellone, Italy

Franklin Forte, Brazil

James Foulds, New Zealand

Igor Francetic, United Kingdom

Anja Frei, Switzerland

Adriano Friganović, Croatia

Ferruccio Galletti, Italy

Ana Gama, Portugal

Yong Gan, China

Ibza García, Mexico

Karin Geffert, Germany

Diana C. Ghanem, Lebanon

Francesco Gianfagna, Italy

Vaitsa Giannouli, Greece

Jenny Godley, Canada

Caroline Goldin, United States

Ana Luiza Gomes Domingos, Brazil

Emanuella Gomes Maia, Brazil

Juan Gómez-Salgado, Spain

Adrián González Marrón, Spain

Alexandre Gouveia, Switzerland

Daniela Grekova-Kafalova, Slovakia

Oliver Gruebner, Switzerland

Calogero Guccio, Italy

Octave Guinebretiere, France

Gabriel Gulis, Denmark

Zoaib Habib Tharwani, Pakistan

Kirubel Tesfaye Hailu, Ireland

Sebastian Haller, Germany

Bing Han, China

Haijing Hao, United States

Jaakko Harkko, Finland

Michael Robert Hässig, Switzerland

Louise Hatherall, United Kingdom

Jan Hattendorf, Switzerland

Margaret Haworth-Brockman, Canada

Yuelin He, United States

Manuel W. Hetzel, Switzerland

Jan Hlodak, Slovakia

Didde Hoeeg, Denmark

Stephanie Hoffmann, Germany

Tekla Hornakova, Czechia

Bernardo Horta, Brazil

Ekhtear Hossain, United States

Bisong Hu, China

Dian Hu, United States

Raghavendra Huchchannavar, India

Daniela Husarova, Bulgaria

Ellen Idler, United States

Elisha Igwe, Nigeria

Federica Intorre, Italy

Christian Gerd Janssen, Germany

Enshe Jiang, China

Aayushi Joshi, Canada

Egide Kalisa, Canada

Elie Karam, Lebanon

Iva Lončarić Kelečić, Croatia

Chitra Khare, United States

Bernard Kikaire, Uganda

Emma Kileel, United States

Elina Kilpi-Jakonen, Finland

Nathan King, Canada

Hana Knezevic Krajina, Croatia

Michaela Kosticova, Slovakia

Rufin Kouassi Assare, Côte d'Ivoire

Jennifer Kowalsky, United States

Levente Kriston, Germany

Nino Kuenzli, Switzerland

Dinesh Kumar, India

Archana Kumari, India

Şerif Kurtuluş, Türkiye

Samantha Lange, South Africa

Rebecca Langella, United Kingdom

Bruno Lankoande, Burkina Faso

Thérésa Lebacq, Belgium

Victoria Leclercq, Belgium

Hsuan-Wei Wayne Lee, Taiwan

Jos Lelieveld, Germany

Roman Andrzej Lewandowski, Poland

Chu-Shiu Li, Taiwan

Yuanyuan Li, China

Karina Lima, Brazil

Bihchuan Lin, Taiwan

Vaclav Linkov, Slovakia

Mohamed Lounis, Algeria

Daniel Ludecke, Australia

Eva Sophie Lunde Pedersen, Switzerland

Valerie Luyckx, Switzerland

Alessandra Macciotta, Italy

Arno Maetens, Belgium

Michelle Magee, United States

Anita Majchrowska, Poland

Altay Manço, Belgium

Erma Manoncourt, France

Raja Marappan, India

Franco Paolo Maray-Ghigliotto, Chile

Mala Mardialina, Indonesia

Francisca Zilania Mariano, Brazil

Raquel Marques Carriço Ferreira, Brazil

Denise Martin, Brazil

Juergen Maurer, Switzerland

Joanna Teresa Mazur, Poland

Kevin Mcintyre, Canada

Neil Mckerrow, South Africa

Mariana Melo, Brazil

Gwenn Menvielle, France

Stéphanie Meynet, Canada

Dragan Mijakoski, North Macedonia

Aukse Miskinyte, Lithuania

Masoud Mohammadnezhad, United Kingdom

Elizabeth Mohr, Germany

Yordanos Molla, United States

Inês Morais Vilaça, Portugal

Francine Müller, Switzerland

Kareem Mumuni, Ghana

Stefano Musumeci, Switzerland

Joanna Myszkowska-Ryciak, Poland

Damalie Nalwanga, Uganda

Nurhasmadiar Nandini, Indonesia

Nhung Nguyen, Vietnam

Huy Nga Nguyen, Vietnam

Patricia Nistor, Canada

Dorit Nitzan, Israel

Matthias Nübling, Germany

Donald Nutbeam, Australia

Zingisa Nyawose, South Africa

Stephen Okumu Ombere, Kenya

Maurice Omollo, Kenya

Elizabeth Orton, United Kingdom

Lyda Osorio, Colombia

Nana Owusu-Boaitey, United States

Esra Özgür, Germany

Veronika Pačutová, Netherlands

Salvatore Panico, Italy

Méryl Paquay, Belgium

Sunghea Park, Switzerland

Upama Paudel, Nepal

Flavia Pennisi, Brazil

Julia Pescarini, United Kingdom

Odessa Petit Dit Dariel, France

Cuong Viet Pham, Vietnam

Cynthia Porter, United States

Caroline Pratt, Georgia

Ivana Prga Borojevic, Croatia

Rodríguez Pulido, Spain

Troy Quast, United States

Faiza Rab, Canada

Rima-Maria Rahal, Austria

Tahereh Rahimi, Iran

Marija Rakovac, Croatia

Arkalgud Ramaprasad, United States

Hasbi Ramdani, Indonesia

Brahim Raouyane, Morocco

Atta Ur Rehman, Pakistan

Slađana Režić, Croatia

Abanoub Riad, Czechia

Heloisa Ribeiro, Portugal

Fulvio Ricceri, Italy

Viviane Richard, Switzerland

Viviane Rodrigues, Brazil

Debra Rog, United States

Domenica Romeo, Italy

Corina Ronneberg, United States

Mathieu Roy, Canada

Emilia Ruggiero, Italy

Bridget Ryan, Canada

Jihene Sahli, Tunisia

Naseem Salahuddin, Pakistan

Diego San José, Spain

Carolina Santos, Portugal

Sofia Santos Pimenta Vale, Portugal

Rodolfo Saracci, France

Abdullah Sarıöz, Türkiye

Alican Sarısaltık, Türkiye

Jasper Schipperijn, Denmark

Andres Ricardo Schneeberger, United States

Diana Schow, United States

Camilla Sculco, Switzerland

Ibrahima Stéphane Séré, Burkina Faso

Florentino Serranheira, Portugal

Rizka Ayu Setyani, Indonesia

Amar Shah, United Kingdom

Akm Shahidullah, Canada

Stav Shapira, Israel

Shubhankar Sharma, Finland

Sukshma Sharma, Italy

Kevin David Shield, Ireland

Pratik Shingru, No Country

Johannes Siegrist, Germany

Dagmar Sigmundová, Czechia

Demsa Simbolon, Indonesia

Vittorio Simeon, Italy

Titiksha Sirari, India

Christine Slade, Australia

C Veronica Smith, United States

Pierre Smith, Belgium

Natasha Sobers, Barbados

Omer Faruk Sonmez, United Kingdom

Nesrine Souayeh, Tunisia

Jana Stein, Germany

Ladislav Stepanek, Czechia

Saverio Stranges, Canada

Pasquale Strazzullo, Italy

Matthias Studer, Switzerland

Suratman Suratman, Indonesia

Dewi Susanna, Indonesia

Alvin Duke Sy, Philippines

Roberto Oscar Tafani, Argentina

Makram Talih, Portugal

Adane Tilahun, Ethiopia

Birhanu Hailu Tirkaso, Ethiopia

Myles Joshua Toledo Tan, United States

Tatiana Toporcov, Brazil

Eni Tresa, Albania

Son Hy Truong, United States

Shangfeng Tsai, Taiwan

Torill Ueland, Norway

Romana Ulbrichtová, Slovakia

Belgin Unal, Türkiye

Jorge Urrutia, Chile

Wells Utembe, South Africa

Maja Vajagic, Croatia

Catharina Francina Van Der Boor, United Kingdom

German Velez, United States

Akarsh Venkatasubramanian, India

Kirsten Verhaegen, Belgium

Giovanni Veronesi, Italy

Marcela Veytia-Lopez, Mexico

Dragana Vidovic, United Kingdom

Paolo Vineis, United Kingdom

Thomas Von Lengerke, Germany

Jörn Von Wietersheim, Germany

Viktor Von Wyl, Switzerland

Nico Vonneilich, Germany

Isidora S. Vujcic, Serbia

Katherine Walesby, United Kingdom

Anqi Wang, United States

Qi Wang, United States

Miriam Wanner, Switzerland

Robert J. Wellman, United States

Jason Were, Canada

Mary Wetani Agoriwo, Ghana

Dorota Weziak-Bialowolska, United States

Frank Wieber, Switzerland

Susan Williams, Australia

Cornelius G. Wittal, Germany

Kyungsook Woo, Republic of Korea

Qiuyi Wu, China

Kaspar Wyss, Switzerland

Ryuichiro Yamamoto, Japan

Fan Yang, China

Iris Zachary, United States

Pavle Zagorscak, Germany

Brian Zanoni, United States

Nejimu Zepro, Switzerland

Ivan Žežula, Slovakia

Jun Zhang, China

Peng Zhang, China

Deshui Zhou, China

Luis Zingg, Netherlands

Pilar Zueras, Spain

